# Gestational Diabetes in the Population Served by Brazilian Public Health Care. Prevalence and Risk Factors

**DOI:** 10.1055/s-0039-1700797

**Published:** 2020-01

**Authors:** Pâmela Antoniazzi dos Santos, José Mauro Madi, Emerson Rodrigues da Silva, Daiane de Oliveira Pereira Vergani, Breno Fauth de Araújo, Rosa Maria Rahmi Garcia

**Affiliations:** 1 Health Sciences Postgraduate Program, Universidade de Caxias do Sul, Caxias do Sul, Rio Grande do Sul, Brazil

**Keywords:** gestational diabetes, public health, prevalence, maternal health, diabetes gestacional, saúde pública, prevalência, saúde materna

## Abstract

**Objective** To assess the prevalence of gestational diabetes mellitus and the main associated risk factors in the population served by the Brazilian Unified Health System in the city of Caxias do Sul, state of Rio Grande do Sul.

**Materials and Methods** A descriptive, cross-sectional and retrospective study was conducted. Maternal variables were collected from the medical records of all pregnant women treated at the basic health units in 2016. Hyperglycemia during pregnancy (pregestational diabetes, overt diabetes and gestational diabetes mellitus) was identified by analyzing the results of a 75-g oral glucose tolerance test, as recommended by the Brazilian Ministry of Health. Based on the data, the women were allocated into two groups: the gestational diabetes group and the no gestational diabetes group.

**Results** The estimated prevalence of gestational diabetes among 2,313 pregnant women was of 5.4% (95% confidence interval [95%CI]: 4.56–6.45). Pregnant women with 3 or more pregnancies had twice the odds of having gestational diabetes compared with primiparous women (odds ratio [OR] = 2.19; 95%CI: 1.42–3.37; *p* < 0.001). Pregnant women aged 35 years or older had three times the odds of having gestational diabetes when compared with younger women (OR = 3.01; 95%CI: 1.97–4.61; *p* < 0.001). Overweight pregnant women were 84% more likely to develop gestational diabetes than those with a body mass index lower than 25 kg/m^2^ (OR = 1.84; 95%CI: 1.25–2.71; *p* = 0.002). A multivariable regression analysis showed that being overweight and being 35 years old or older were independent variables.

**Conclusion** In this population, the prevalence of gestational diabetes mellitus was of 5.4%. Age and being overweight were predictive factors for gestational diabetes.

## Resumo

**Objetivo** Avaliar a prevalência de diabetes mellitus gestacional, e dos principais fatores de risco associados, em população usuária do Sistema Único de Saúde em Caxias do Sul-RS.

**Métodos** Um estudo descritivo, transversal e retrospectivo foi feito. As variáveis maternas foram coletadas de registros de prontuários de todas gestantes atendidas nas Unidades Básicas de Saúde do município em 2016. A identificação de hiperglicemia na gestação (diabetes pré-gestacional, diabetes identificado durante a gestação e diabetes mellitus gestacional) foi feita pela avaliação dos resultados do teste oral de tolerância com 75 g glicose, conforme preconizado pelo Ministério da Saúde. Com base nesses dados, as gestantes foram separadas em dois grupos: o grupo com diabetes gestacional e o grupo sem diabetes gestacional.

**Resultados** A prevalência estimada de diabetes gestacional em 2.313 gestantes foi de 5,4% (intervalo de confiança de 95% [IC95%]: 4,56–6,45). Gestantes com 3 ou mais gestações apresentaram chance 2 vezes maior para a ocorrência de diabetes gestacional, quando comparadas às primigestas (razão de possibilidades [RP] = 2,19; IC95%: 1,42- 3,37; *p* < 0,001). Gestantes com idade de 35 anos ou mais apresentaram chance três vezes maior do que as mais jovens (RP = 3,01; IC95%: 1,97–4,61; *p* < 0,001). A chance de desenvolver diabetes gestacional em gestantes com sobrepeso foi 84% maior do que a das com índice de massa corporal inferior a 25 kg/m^2^ (RP = 1,84; IC95%: 1,25–2,71; *p* = 0,002). A análise de regressão multivariada mostrou sobrepeso e idade materna como variáveis com associação independente.

**Conclusão** Nesta população, a prevalência de diabetes mellitus gestacional foi de 5,4%. Idade materna e sobrepeso pré-gestacional foram fatores preditivos para diabetes gestacional.

## Introduction

In the past 20 years, the global epidemic of diabetes and obesity has reached the population of women of reproductive age; in parallel, there was an increase in the incidence of hyperglycemia during pregnancy.^1^ ^2^ The International Diabetes Federation estimated that, in 2017, 21.3 million (16.2%) live births were from pregnancies with hyperglycemia; 86.4% of these were due to gestational diabetes mellitus (GDM), 6.2% were due to diabetes detected before pregnancy, and 7.4% were due to other types of diabetes (including type-1 and type-2 diabetes) detected for the first time during pregnancy.^3^

This broad variation results from multiple of methodological issues, such as the absence of universal criteria for GDM screening and different population characteristics.^2^ In addition, there are little data available regarding estimates of the global prevalence of GDM, especially in developing countries.

In 2016, Zhu and Zhang^4^ reported a large variation in the prevalence of GDM in different regions of the world, including a higher prevalence in the Middle East and North Africa (12.9%), Southeast Asia (11.7%) and regions of the Western Pacific (11.7%), and a lower prevalence in Europe (5.8%). In Central and South America, the prevalence was of 11.2% (95% confidence interval [95%CI]: 7.1–16.6); however, this figure was derived based on data from only two countries: Brazil (5.7%) and Cuba, (16.6%). Brazil has few studies regarding this issue,^5^ ^6^ ^7^ the most relevant being that of Schmidt et al (2001),^5^ which showed an estimated prevalence between 2.4% and 7.2%, depending on the criteria used to diagnose GDM.

Transitional hyperglycemia during pregnancy complicated by GDM occurs primarily due to the functional incapacity of maternal β-pancreatic cells to meet the insulin needs for adequate fetal development; this insufficiency is accentuated starting in the second gestational trimester.^8^ This metabolic complication is associated with long-term perinatal and long-term outcomes for the maternal-fetal pair,^9^ ^10^ such as excessive fetal growth and consequent complications during labor.^9^ A history of GDM in pregnancy is associated with a higher risk of metabolic syndrome, type-2 diabetes mellitus (DM2) and cardiovascular diseases in postchildbirth follow-ups.^10^ Approximately 50% of women with GDM progress to DM2 after 10 years.^10^

Gestational diabetes mellitus can have a bigger impact on the health of the mother and her offspring, and it is suggested that it plays a significant role in the global diabetes epidemic. While its prevalence has increased in different populations throughout the world in recent decades, individual reports of this global trend cannot be compared because of the variety of methodological issues. Nonetheless, GDM is an important public health problem today, and it affects the heterogeneous Brazilian population. Considering the relevance of this topic, the present study was developed to estimate the prevalence of GDM and evaluate the associated risk factors among the users of the Brazilian Unified Health System in the city of Caxias do Sul, state of Rio Grande do Sul.

## Materials and Methods

A cross-sectional, retrospective, prevalence study was performed from January 1st to December 31st, 2016, in a population of pregnant women who were users of the Unified Health System (UHS) and attended prenatal follow-up visits at the 47 basic health care units (BHCU) in the city of Caxias do Sul. The present study was approved and supported by the Municipal Health Department and by the Research Ethics Committee of Universidade de Caxias do Sul (number: 2,048,666).

A total of 3,411 medical records were searched for the selected period, which were registered in the SisPreNatal-Datasus software of the Brazilian Ministry of Health and filed in their respective health care units. The medical records that were not found after numerous attempts throughout the entire period of data collection were considered lost. All of the evaluated medical records were from pregnant women residing in Caxias do Sul. Demographic, clinical, and laboratory data were collected and transferred to a database designed for the study and handled exclusively by the researcher responsible for it. The following variables were collected: mother's age (years); race/ethnicity (Caucasian, of African descent and other); level of schooling (≤ 8 years and > 8 years); family history of diabetes in first-degree relatives; obstetric history of GDM; previous hypertensive syndrome, defined as hypertension, preeclampsia and eclampsia; previous abortions; smoking during pregnancy; parity (1, 2 or ≥3); pregestational weight (kg) obtained at the first prenatal visit; height (centimeters); and pregestational body mass index (BMI). Fasting glycemia, glycated hemoglobin, and/or 75-g oral glucose tolerance test (OGTT) results and/or the use of antihyperglycemic drugs were analyzed to identify the presence of hyperglycemia during pregnancy. To identify GDM in the study population, the OGTT results were analyzed, as recommended by the Brazilian Ministry of Health,^11^ and a positive diagnosis was made when one or more of the following criteria were present: glycemia (fasting) ≥ 92 mg/dl and ≤125 mg/dl; blood glucose 1 hour after overload ≥180 mg/dl; glycemia 2 hours after overload ≥153 mg/dl and ≤ 199 mg/dl. Pregestational diabetes or overt diabetes was considered if glycemia (fasting) ≥ 126mg/dl and/or glycemia 2 hours postoverload ≥ 200 mg/dl and/ or glycated hemoglobin > 6.5%.^11^

The statistical analysis of the data was performed through univariate and multivariate logistic regression, using GDM as a variable response. The variables included in the multivariate regression were selected by the backward technique if they presented a *p*-value < 0.15 in the univariate step and the percentage of missing data was lower than 10%.^11^

The R software (R Foundation, Vienna, Austria) was used in the statistical analysis of the data. The presence of multicollinearity was evaluated by the estimation of variance inflation factors (VIFs); VIF values > 2.5 indicated considerable multicollinearity in the logistic regression analysis. The calibration and discriminatory ability of the final multiple logistic regression model were evaluated using the Hosmer-Lemeshow test. Values of *p *≥ 0.05 for the Hosmer-Lemeshow test indicated which of the models was calibrated.^12^

## Results

There were 3,411 medical records registered by the Health Department of those, 2,797 (82%) were filed and therefore available for the study. Of these 2,797 filed records, 484 (17%) had no clinical or laboratory data to identify any type of hyperglycemia or glycemic normality during the gestational period; therefore, they were excluded from the study; 2,313 records contained fasting glycemia information at the first visit; 1,079 had OGTT information; no charts contained information on glycated hemoglobin; 856 had information on another type of fasting glycemia; 1,079 medical records contained fasting glucose at the first visit and OGTT, and 1,234 did not provide OGTT data.

Thus, the study sample consisted of 2,313 medical records. Based on the data collected from these charts, the patients were allocated into two groups: 1) pregnant women without GDM (2,187; 94.6%) and 2) pregnant women with GDM (126; 5.4%).

**Fig. 1:**
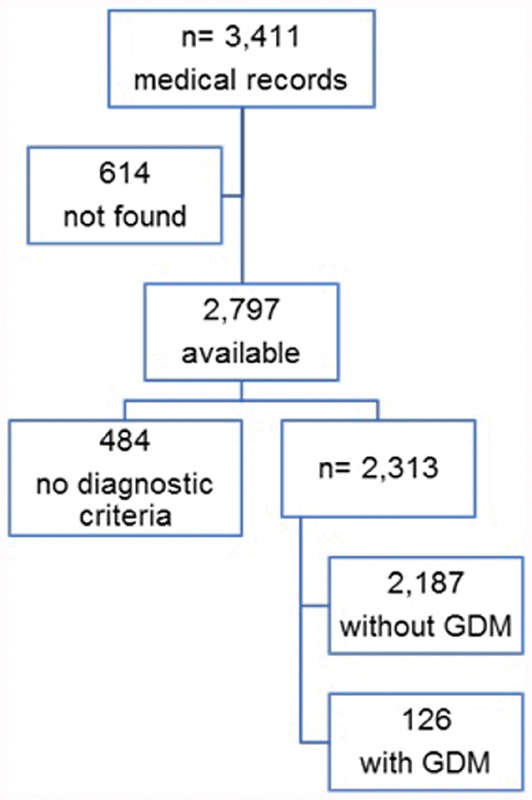
Flowchart for the composition of the groups with and without gestational diabetes mellitus (GDM) based on 3,411 medical records of pregnant women.

In group 1, 25 (1.1%) records met the criteria for pregestational DM, but did not meet the criteria for GDM. The 126 charts that composed group 2 met the criteria for GDM based on OGTT results (Fig. 1). The estimated prevalence of GDM was of 5.4% (95%CI: 4.56–6.45).

The analysis of the variables showed that pregnant women aged ≥ 35 years were three times more likely to develop GDM than younger women (OR = 3.01; 95%CI: 1.97–4.61; *p* < 0.001) (Table 1). Pregestational BMI ≥ 25 kg/m^2^ doubled the chance of developing GDM compared with a lower BMI (OR = 1.84; 95%CI: 1.25–2.71; *p* = 0.002) (Table 2). Women with 3 or more pregnancies had 2 times higher odds of having GDM than primiparous women (OR = 2.19; 95%CI: 1.42–3.37; *p* < 0.001). The likelihood of women with 2 pregnancies developing GDM was not statistically significant (OR = 1.19; 95%CI: 0.72–1.98; *p* = 0.503) (Table 2).

**Table 1:** Sociodemographic characteristics of pregnant women with and without gestational diabetes mellitus

Variable	Group 1 n (%)	Group 2 n (%)	Odds ratio (95% confidence interval)	*p*-value
Age (years)				
<35^a^	1,939 (95.4)	94 (4.6)		
≥35	219 (87.3)	32 (12.7)	3.01 (1.97–4.61)	< 0.001
Race/Ethnicity				
Caucasian^a^	589 (94.8)	32 (5.2)		
Of African descent	49 (89.1)	6 (10.9)	2.25 (0.90–5.65)	0.083
Other	83 (94.3)	5 (5.7)	1.11 (0.42–2.93)	0.835
Schooling (years)				
≤8^a^	748 (94.9)	40 (5.1)		
> 8	826 (94.1)	52 (5.9)	1.18 (0.77–1.80)	0.451

Notes: ^a^Reference category. Group 1: no GDM; group 2: with GDM.

**Table 2:** Clinical characteristics of the groups of pregnant women with and without gestational diabetes mellitus

Variables	Group 1 n (%)	Group 2 n (%)	Odds ratio (95% confidence interval)	*p*-value
Pregestational BMI (kg/m^2^)				
≤24.9^a^	1,043 (96)	43 (4)		
>25	949 (92.9)	72 (7.1)	1.84 (1.25- 2.71)	0.002
Parity				
1	886 (92.2)	35 (3.8)		
2	596 (95.5)	28 (4.5)	1.19 (0.72- 1.98)	0.503
3	670 (92)	58 (8)	2.19 (1.42- 3.37)	< 0.001
Smoking				
No^a^	1539 (95)	81 (5)		
Yes	309 (93.1)	23 (6.9)	1.41 (0.88- 2.28)	0.156
PHS				
No^a^	1945 (94.6)	110 (5.4)		
Yes	53 (93)	4 (7)	1.34 (0.47- 3.76)	0.584
Previous GDM				
No^a^	1981 (94.8)	108 (5.2)		
Yes	17 (85)	3 (15)	3.24 (0.93–11.21)	0.064
Previous abortion				
No^a^	1792 (94.9)	97 (5.1)		
Yes	240 (92.3)	20 (7.7)	1.54 (0.93- 2.54)	0.091
AF-DM2				
No^a^	1321 (95.3)	65 (4.7)		
Sim	563 (93.4)	40 (6.6)	1.44 (0.96- 2.17)	0.076
Height (cm)				
≤150^a^	140 (6.7)	7 (5.8)		
>150	1945 (93.3)	113 (94.2)	1.16 (0.53- 2.54)	0.707

Abbreviations: AF-DM2, family history of type-2 diabetes mellitus; BMI, body mass index; GDM, gestational diabetes mellitus; PHS, previous hypertensive syndromes (hypertension, preeclampsia and eclampsia).

Notes: ^a^Reference category. Group 1: no GDM; group 2: GDM.

Women of African descent (OR = 2.25; 95%CI: 0.90–5.65; *p* = 0.083) and those classified as being of another race/ethnicity (OR = 1.11 95%CI: 0.42–2.93; *p* = 0.835), those with higher levels of schooling (< 8 years; OR = 1.18; 95%CI: 0.77–1.80; *p* = 0.451), those who smoked during pregnancy (OR = 1.41; 95%CI: 0.88–2.28; *p* = 0.156), and those with previous hypertensive syndromes (PHSs; OR = 1.34; 95%CI: 0.47–3.76; *p* = 0.584), previous GDM (OR = 3.24; 95%CI: 0.93–11.21; *p* = 0.064), previous abortion [ 93- 2.54), *p* = 0.091], family history of type-2 diabetes mellitus (AF-DM2; OR = 1.44 95%CI: 0.96–2.17; *p* = 0.076) and low height (OR = 1.16 95%CI: 0.53–2.54; *p* = 0.707) did not have an increased likelihood of developing GDM compared with the reference categories (Tables 1 and 2).

Age ≥ 35 years and BMI ≥ 25 kg/m^2^ were independent variables with a significance level of 5%. Hosmer-Lemeshow statistics indicated that the logistic model was satisfactorily adjusted, with agreement between the observed and the expected frequencies of the outcome (*p* = 0.794). The area under the receiver operating characteristic (ROC) curve (AUC) associated with the multiple logistic regression model was 0.62. Therefore, the model had an almost perfect performance to discriminate between the categories of the binary outcome (whether someone has or does not have GDM) (Table 3).

**Table 3:** Results of the binary multiple logistic regression analysis

Variable	Odds ratio (95% confidence interval)	*p*-value^b^
Age (years)		
<35^a^		
≤35	3.124 (1.904–5.125)	< 0.001
Body mass index (kg/m^2^)		
≤24.9^a^		
≥25	1.498 (0.966–2.324)	0.0711

Notes: N = 1785. ^b^ *p* = 0.794 according to the Hosmer-Lemeshow test (calibration); ^a^reference category; area under the curve (AUC) = 0.62 (discrimination).

## Discussion

The present study showed that 5.4% of pregnant women cared for in 2016 by the Unified Health System in Caxias do Sul had GDM. In this population, women who became pregnant and were overweight/obese had the most frequent metabolic complications during pregnancy. Despite the methodological differences, the results of the present study showed a certain similarity to those described by Schmidt et al^5^ in 2001, who estimated the prevalence of GDM based on data from six Brazilian capitals (Porto Alegre, São Paulo, Rio de Janeiro, Salvador, Fortaleza and Manaus). In that study, the authors concluded that the prevalence of GDM was of 2.4% (95%CI: 2.0–2.9) according to the American Diabetes Association (ADA) 2000 diagnostic criteria and of 7.2% (95%CI: 6.5–7.9) according to the World Health Organization (WHO) 1999 criteria.

Studies show that the number of pregnant women with GDM has been increasing in recent decades in a proportion parallel to that of DM2.^2^ ^3^ This scenario requires effective commitment from all health areas involved with women's health during pregnancy. When analyzing the prevalence of GDM in different global regions, the results vary according to ethnic/racial, socioeconomic and cultural characteristics and screening criteria.^13^ ^14^ India has observed a significant increase in the prevalence of GDM, with large differences among regions.^15^ This situation led the WHO (2016),^16^ to implement the pilot project “The Women in India with GDM Strategy (WINGS)” with the aim of developing a suitable model of care for women with GDM in low-and middle-income countries.

We have scarce scientific literature showing the epidemiological reality of GDM in Brazil. The most relevant and comprehensive study on GDM was published in 2001.^5^ During this period of nearly 20 years, population, health and socioeconomic indicators underwent significant changes, and epidemiological transitions occurred in a peculiar way.^17^ National epidemiological studies designed with a targeted objective could provide evidence-based information about the current reality and trends of GDM. The present study was developed at Universidade de Caxias do Sul in partnership with the Municipal Health Department to obtain a more in-depth picture of pregnant women with GDM. These patients are referred from the BHCUs to the High-Risk Pregnancy Clinic of the university, and GDM is the main cause for referral to this secondary/tertiary care unit. In addition to providing assistance, this clinic offers long-term, multidisciplinary follow-up for these women and their offspring, and has both academic and care provision goals.

The racial/ethnic characteristics of the local population are quite homogeneous; 82.52% are Caucasians,^18^ the majority of whom are descendants of Italian immigrants who settled in the state of Rio Grande do Sul in the second half of the 19th century. Our results are pertinent to a specific reality of the southern region of Brazil, and therefore they might differ from those of other regions. The results did not show significant differences in the likelihood of developing GDM among ethnic subgroups (Caucasians, those of African descent, and those of other ethnicities); however, our findings were discordant with those presented by Hedderson et al,^13^ who identified a variation in the risk of developing GDM among different racial groups within and outside the United States, in a retrospectively examined multiethnic population of 216,089 pregnant women.

Women with the most advanced maternal age (≥35 years) had twice as much chance of developing GDM than younger women. Concordant results were found in a study conducted in the city Pelotas, state of Rio Grande do Sul, in 2009, in which a population of 4,243 women older than 35 years showed an OR of 6.09 for GDM in late pregnancy, compared with younger pregnant women (age: < 20 years).^6^ In this context, we highlight a study by Lao et al^19^ in a population of 15,827 primiparous women, who showed a progressive increase in the risk of developing GDM with increasing maternal age, starting at 25 years. In recent years, there has been a significant increase in the number of women who become pregnant at the age of 35 years or older. This increase was of 28% between 2010 and 2016 in Brazil; in the region of the present study, the increase was of 27%.^20^ The surveillance system for risk factors and protection for chronic diseases based on telephone survey (VIGITEL, in Portuguese), estimated in 2017 that among women aged > 18 years, there was an increase in BMI as age advanced.^21^ In addition, it is worth noting the lack of knowledge among the female population regarding the risks of gestation at a later age.^22^ This combination of important risk factors for GDM deserves special attention from public health managers to implement prevention programs.

A high percentage (48%) of overweight (BMI ≥ 25 kg/m^2^) women was identified in the present study, which is in agreement with the 2017 VIGITEL report,^21^ which stated that 51.2% of women aged ≥ 18 years were overweight.^21^ This finding corroborates other results that describe a linear increase in the cases of GDM as the maternal BMI increases.^6^ ^23^ ^24^

The number of pregnancies has been evaluated as a non-traditional risk factor for the development of GDM. Parity ≥3 resulted in a greater chance of developing GDM compared with primiparous women (*p* < 0.001), but this association lost significance in the adjusted analysis. Jesmin et al,^25^ in a study with 3,447 pregnant women in Bangladesh, reported a higher risk of GDM with increased gestation numbers.^25^ However, Seghieri et al^26^ did not identify a direct association of this variable with the progression of pancreatic cell dysfunction and the onset of GDM, and suggested obesity and maternal age as possible mediating factors.

A family history of DM2, level of schooling, smoking, previous hypertension and low maternal height showed no association with the outcome in the present study. However, careful interpretation of these results is necessary due to the methodological limitations inherent to the retrospective design. Data collection from medical records can be challenging because numerous clinical data are not filed despite being fundamental to qualified medical care and for scientific research. This serious problem has existed for decades, but could be improved by the introduction of standardized, digitalized medical records containing a minimum number of mandatory information fields. In addition, there is a need for permanent monitoring, which could be performed using sample surveys on six-month basis. The Brazilian Ministry of Health and the state and municipal health departments have engaged in dialogue regarding the adoption of measures to improve their data records.

Despite the limitations of the present study, the extensive bibliography sometimes corroborated our findings; however, at other times, the literature refuted our results, which were sometimes controversial.^5^ ^6^ ^23^ ^27^ ^28^ ^29^ ^30^ The different results indicate that more studies are needed to establish the real association of various factors with GDM.

## Conclusion

The present study analyzed maternal age as predictive factor for GDM in the population of pregnant women who are users of the Brazilian Unified Health System in the city of Caxias do Sul. Despite the limitations described, the collected data came from a sample that represents about ∼ 50% of the population of pregnant women who attended health care services in the city in 2016. This epidemiological study provided a qualitative and quantitative evaluation of the information contained in medical records, and highlighted the insufficient logistics involved in filing such records, which could be used by the city hall managers and staff. The results of the present study describe a complication of pregnancy, and may be a starting point for more comprehensive prospective studies in the near future. To conclude, population-based scientific research in accordance with regional needs should be promoted because the university-community partnership tends to strengthen the production of knowledge and integrate the theoretical content of academic disciplines with practical reality, which can result in substantial scientific development.
